# Methylation Markers for the Identification of Body Fluids and Tissues from Forensic Trace Evidence

**DOI:** 10.1371/journal.pone.0147973

**Published:** 2016-02-01

**Authors:** Sophia Forat, Bruno Huettel, Richard Reinhardt, Rolf Fimmers, Gerhard Haidl, Dominik Denschlag, Klaus Olek

**Affiliations:** 1 Labor für Abstammungsbegutachtungen GmbH, Rheinbach, Germany; 2 Max Planck Genome Centre Cologne Institute for Breeding Research, Cologne, Germany; 3 Institute for Medical Biometry, Informatics and Epidemiology, University of Bonn, Bonn, Germany; 4 Department of Dermatology, Andrology Unit, University of Bonn, Bonn, Germany; 5 Department of OB/GYN Hochtaunuskliniken Bad Homburg, Bad Homburg, Germany; Chung-Ang University, REPUBLIC OF KOREA

## Abstract

The identification of body fluids is an essential tool for clarifying the course of events at a criminal site. The analytical problem is the fact that the biological material has been very often exposed to detrimental exogenous influences. Thereby, the molecular substrates used for the identification of the traces may become degraded. So far, most protocols utilize cell specific proteins or RNAs. Instead of measuring these more sensitive compounds this paper describes the application of the differential DNA-methylation. As a result of two genome wide screenings with the Illumina HumanMethylation BeadChips 27 and 450k we identified 150 candidate loci revealing differential methylation with regard to the body fluids venous blood, menstrual blood, vaginal fluid, saliva and sperm. Among them we selected 9 loci as the most promising markers. For the final determination of the methylation degree we applied the SNuPE-method. Because the degree of methylation might be modified by various endogenous and exogenous factors, we tested each marker with approximately 100 samples of each target fluid in a validation study. The stability of the detection procedure is proved in various simulated forensic surroundings according to standardized conditions. We studied the potential influence of 12 relatively common tumors on the methylation of the 9 markers. For this purpose the target fluids of 34 patients have been analysed. Only the cervix carcinoma might have an remarkable effect because impairing the signal of both vaginal markers. Using the Illumina MiSeq device we tested the potential influence of cis acting sequence variants on the methylation degree of the 9 markers in the specific body fluid DNA of 50 individuals. For 4 marker loci we observed such an influence either by sole SNPs or haplotypes. The identification of each target fluid is possible in arbitrary mixtures with the remaining four body fluids. The sensitivity of the individual body fluid tests is in the same range as for the forensic STR-analysis. It is the first forensic body fluid protocol which considers the exogenic and endogenic parameters potentially interfering with the true results.

## Introduction

The analysis of a biological trace found at the crime site ought to help to identify the perpetrator and to clarify the course of events. The most efficient tool for getting information about the identity of the involved persons is the genetic fingerprint, whereas the biological nature and composition of the material very often elucidates the course of events. In contrast to the very clear and world wide standardized situation for the genetic fingerprint, the identification of body fluids from forensic traces is still a severe problem. Typically, a biological trace at the crime site is exposed to various physical, biochemical and microbiological impacts, most of them finally destroying the biological components that are being used to identify a body fluid. A widely used procedure is the stepwise analysis of the material [1–5]. Unfortunately, the unspecific tests applied as a first step require much of the sample material or even destroy the DNA which is necessary for the genetic fingerprint. But more importantly, almost all available subsequently used specific tests are insufficient. Juusola et al describe a procedure that bases on the mRNA technique. The authors utilize the tissue specific expression of the STATH and HTN3 genes [6]. The usage of micro RNAs represents another promising approach to solve the described problems [7, 8]. Considering the usual condition of forensic biological material, the application of the cell or tissue specific methylation as a tool for discrimination and identification of body fluids seems to be most attractive, because the analytical substrate is the DNA, which is rather stable compared to any type of RNA and protein. Furthermore, the expression of most RNA markers overlaps between single body fluids. In the past three years essentially two groups tried to use methylation to detect specific body fluids [9, 10, 11]. Many methylated loci are involved in the long term regulation of the cell specific gene expression. Thus, a cell line may be precisely characterized by quantifying the degree of methylation at such positions [12]. Most body fluids contain various different cell types, which makes the usage of cell specific methylation a challenging approach. According to this cellular heterogeneity, additional problems may arise as this cell composition eventually changes due to changing metabolic stages. Whereas the DNA itself is surprisingly stable even if exposed to extreme exogenous stress, we know only little about the stability of the methylated DNA-body. The genomic methylation pattern of an individual is remarkably dependent on its age and maybe on its lifestyle [13, 14]. Cancer diseases are known to influence the methylation pattern of the affected patients. Sequence variants are able to modify the degree of methylation in cis positioned CpGs. As described for numerous phenotypes the sequence variants may exert their function as Quantitative Trait Loci (mQTL) [15, 16] or as allele specific methylation (ASM) [17, 18]. These parameters might have a significant impact on the reliability of a methylation-based procedure for forensic body fluid analysis.

This study describes an epigenetic marker set detecting venous blood, menstrual blood, vaginal fluid, saliva and sperm from forensic material. It was the aim of this work to develop a test system which considers all the above mentioned factors potentially affecting the reliability of the test. For this purpose we performed two genome wide chip based studies (Illumina 27k, 450k) in order to get an appropriate number of differentially methylated loci. From these initial screening experiments we obtained many candidates among which we selected 9 marker loci specific for peripheral blood, menstrual blood, vaginal fluid, saliva and sperm. Using the SNuPE technology, we performed an extended validation-study to characterize the distribution of the methylation values. Furthermore, the body fluids were exposed to various standardized environmental conditions, thus simulating forensic situations [19]. We analyzed the body fluids with regard to our methylation markers of 34 probands suffering from 12 different relatively common tumors. Using the NGS-technology, we tried to identify any potential cis-influence of genetic variants on the methylation state of the linked CpGs we utilized in our protocols. We verified the applicability of the procedure in detecting the target body fluids from mixtures. To our experience the SNuPE-method is a reasonable compromise, which provides for the fact that we deal with a quantitative trait regarding the methylation and that the method should be feasible in a normal forensic genetic laboratory.

## Materials and Methods

### Genome-wide DNA Methylation Profiling and Marker Selection

Two genome wide methylation studies were carried out on Illumina Human Methylation BeadChips 27 and 450k at intervals of three years. The more recent HumanMethylation450 BeadChip assays DNA methylation at 482,421 CpG sites, including 90% of the 27,578 sites of the 27K array. Hence, the second study was performed in order to achieve even more marker candidates. For both methylation screenings different samples of every target fluid were used. In each study two DNA-samples (500ng) of peripheral blood, menstrual blood, saliva, vaginal fluid, sperm, and one sample of endometrium, semen of vasectomized men, skin and penis mucosa, respectively, were analyzed. For each sample of body fluid we pooled equal amounts of DNA from 3–8 individuals in order to correct the interindividual variability of methylation. DNA was extracted and bisulfite converted using standard protocols (DNeasy Blood & Tissue Kit, EpiTect^®^ Bisulfite Kit, Qiagen). The microarray hybridization and extraction of the β-values was performed following the manufacturer´s instructions (Illumina). The β-value corresponds to the percentage of methylation at each detected CpG site and varies between 0 (no methylation) and 1 (100% methylation). The best discriminating sites were searched by comparing the average methylation values of both target body fluid samples to every other sample respectively. The candidate CpG sites should exhibit β-value of at least 0,4 in the target fluid whereas the ß-values of the remaining samples should not exceed 0,05. In order to identify reciprocal marker loci—the target is unmethylated and the remaining fluids are methylated—we applied the same thresholds, but the other way around. The CpGs with the highest discriminant power were selected as marker candidates. For each candidate site we designed PCR primers for the amplification of bisulfite converted genomic DNA to generate amplicons for the sequencing. The obtained data were used to select CpGs adjacent to the ones identified by the chip-analysis with—if possible—even more discriminating power than these. In fact, for the most loci we did not apply the original CpGs for the final msSNuPE analysis.

### Sample Collection

For the statistical validation a total of 100 peripheral blood (45 female, 55 male), 96 menstrual blood, 100 saliva (56 female, 44 male), 55 vaginal fluid and 91 sperm samples were collected. In most cases menstrual blood and vaginal fluid samples were supplied from the same female donors. A total of 197 female and 190 male donors provided written informed consent, after the background of the study had been explained. The project was approved by the local Ethics committee in Bad Homburg vdH, Germany and the Ethics committee of the University of Bonn, Germany. Saliva and vaginal fluid were provided on sterile cotton swabs. Fresh peripheral blood and sperm were collected in microcentrifuge tubes. Menstrual blood was provided on tampons or sanitary napkins. Furthermore we wanted to check the cross reaction of the markers with other body fluids and tissues which sometimes could also be part of a mixture on a crime scene. For this purpose we collected two samples of urine (one male, one female), two samples of semen derived from vasectomized men and 4 samples of dander derived from scalpel and forearm of male and female persons.

The anonymous carcinoma samples were provided by 34 patients (13 men and 21 women) from various clinical institutions in Bad Homburg and the University Hospitals in Bonn. The following samples were obtained: 7 blood samples of CLL (chronic lymphocytic leukemia), 3 blood samples of CML (chronic myelocytic leukemia), 1 blood sample of MPS (myeloproliferative syndrome), 2 blood samples of B-cell lymphoma, 9 blood samples of patients with breast carcinoma, 3 blood samples of patients with colorectal carcinoma (one of poliposis adematosa familiar and two of hereditary nonpolyposis colorectal cancer), 2 sperm samples of patients with testicular cancer, 1 saliva sample of a patient with esophagus carcinoma, 1 vaginal fluid sample of a patient with endometrial carcinoma, 2 vaginal fluid samples of patients with cervix carcinoma, 2 vaginal fluid samples of patients with ovarian carcinoma and 1 sample of a patient with endometriosis. All samples were stored frozen until being processed.

### Sample Preparation for Forensic Validation

The forensic simulation experiments were arranged according to the recommendations of Setzer et al [19]. 50μl of peripheral blood, menstrual blood and sperm were applied on pieces of cotton. Vaginal fluid and saliva were stored on cotton swabs. To simulate the forensic reality three kinds of environmental influences were prepared—dry on room temperature, wet in an exsiccator and outside on the ground. The initial analysis was done immediately (t_0_) after sample taking. Subsequently, the samples were tested after 1, 2, 3 and 6 months of storage. Thus, a total of 75 body fluid samples were prepared and analyzed for the forensic validation.

### Processing of Body Fluid Samples

DNA was extracted from blood, saliva and urine using a QIAmp^®^ DNA Mini Kit (Qiagen) and from menstrual blood, vaginal swabs, sperm, semen of vasectomized men and skin using a DNeasy^®^ Blood & Tissue Kit (Qiagen) following manufacturer´s instructions. For the extraction of sperm additional 20μl 1M DTT were used. For statistical validation 100μl peripheral blood, 50μl sperm, a piece (0,5cm^2^) of a tampon or sanitary napkin and one swab of vaginal fluid and saliva respectively were used. For the forensic validation 1/2 spot of peripheral blood, menstrual blood and sperm stains, as well as 1/2 swab of vaginal fluid and saliva stains were used.

The DNA extracts were quantified using the Nanodrop^™^ ND-1000 Spectrometer (NanoDrop Technologies) and stored frozen until processing. The bisulfite conversion of DNA was carried out using a EpiTect^®^ Plus Bisulfite Kit and EpiTect^®^ 96 Bisulfite Kit (Qiagen) according the manufacturer´s protocols. For validation experiments 2ng DNA were used. In forensic experiments the total amount of the extracted DNA was converted. For the sensitivity test variable quantities of DNA were applied to get final concentrations of 5ng to 25pg bisulfite converted DNA per μl.

### Preparation of Mixtures

In the forensic reality body fluid stains are often mixtures. To study the potential impact of admixed body fluids on the methylation marker of the target we analyzed each marker in mixtures containing 0, 20, 40, 60, 80 and 100 percent DNA of the marker specific fluid and 100, 80, 60, 40, 20 and 0 percent of an equimolar mixture of the remaining 4 body fluid DNAs. Each mixed sample and each pure sample was quantified and bisulfite converted prior to the analysis.

### PCR-Conditions

One μl of bisulfite converted DNA was amplified in a final volume of 25μl. Two different PCR-mixtures were carried out. The amplicons Blut1, Blut2, Spei1, Spei2, Vag1, Vag2 and Sperm1 were amplified in a reaction containing 1x PCR Buffer, 2.0 mM MgCl, 0.2mM dNTP,1U Taq DNA polymerase (Qiagen) and 4,5pmol each of forward and reverse primers (S1 Table). The amplicons Mens1 and Sperm2 were amplified without MgCl. The amplification conditions were 95°C for 15 min, 10 cycles of 95°C for 1 min, 56°C for 45 sec and 72°C for 1min, 20 cycles of 95°C for 1 min, 55°C for 45 sec and 72°C for 1min and 10 cycles of 95°C for 1 min, 54°C for 45 sec and 72°C for 1min, and a final extension step of 10 min at 72°C. PCR products were purified using ExoSAP IT^®^ (USB).

### Targeted Bisulfite Sequencing

Sequencing reaction was accomplished using a modified Sanger sequencing protocol. Purified PCR products were sequenced applying 1pmol forward or reverse PCR primer, 25μM dNTP and the ABI Big Dye Terminator v1.1 cycle sequencing chemistry (Applied Biosystems) followed by capillary electrophoresis on an ABI 3730 genetic analyzer. Sequencing files were interpreted using ESME [20]. This software allows for quantification of methylation signals after normalizing the traces and correcting for incomplete bisulfite conversion.

### Methylation Sensitive Single Nucleotide Primer Extension Assay (msSNuPE)

The assay utilizes internal primers annealing straight 5´ of the nucleotide to be detected. The primer is extended by one complementary fluorescent labeled ddNTP (S1 Fig). To anneal on both sequence types—methylated and unmethylated—the target sequence of the primer may not contain further CpGs. Due to the high CpG density we applied a slightly different detection strategy for Mens1 and Spei1. The primer sequence covers two CpGs, which may be methylated or unmethylated. Here, we simultaneously used one primer completely matching to the methylated sequence the other one to the unmethylated (S2 Fig). The primers end 5’ with a different nucleotide. For Blut1 and Blut2 we used two different CpGs as diplex primer extension assay.

MS-SNuPE was carried out using the ABI SNaPshot Multiplex Kit (Applied Biosystems). Sample DNA was amplified using the PCR conditions mentioned in the main paper Materials and Methods. The reaction was performed in 10μl volume containing 1pmol internal SNuPE-Primers (S2 Table) and 3μl purified PCR products. After thermal cycling the extension products were treated with 1U Shrimp Alkaline Phosphatase (Thermo Fischer scientific). Analysis was performed by capillary electrophoresis using ABI 3730 Genetic Analyzer and GeneMapper ID software (v3.2). For quantifying the methylation we determined the ratio of methylated/unmethylated fluorescence signal and the sum of both, thereby circumventing the semiquantitative PCR-results.

### Next Generation Sequencing (NGS)

Because we applied the SNuPE-assay, bisulfite sequencing, the 454-pyrosequencing (Roche Diagnostics) and the MiSeq (Sequencing-by-synthesis, Illumina) for various quantifying experiments we compared their relative analytical efficiency. For this comparison experiment the same DNA samples of each body fluid were used.

To elucidate possible genetic influences on the methylation degree we sequenced our analytical CpGs and their molecular surrounding only on the MiSeq-platform. For that purpose we studied all of the 5 body fluids. For each type of body fluid we performed 5 PCRs containing equimolar amounts of pooled bisulfite converted DNA from 10 individuals respectively. So altogether, we sequenced the methylation loci from 50 individuals for each body fluid.

For both experiments the corresponding samples were amplified with above mentioned PCR-protocol. We constructed a single library using forward and reverse primers of the 9 loci provided with 18 different barcodes. PCR products were purified with magnetic beads (Beckman Coulter) and fluorometrically quantified. For 454-pyrosequencing we used the rapid library preparation method (Roche) according to the manufacturer's instructions for the 454 GS FLX+ platform. Equimolar amounts of the MID-generated amplicons were pooled to a library and sequenced on a 454 Genome Sequencer FLX+ System following the amplicon sequencing protocol with GS Titanium chemistry. For validation of the 454 data only complete reads with a quality score above 20 were used.

For the NGS analysis on the MiSeq-instrument the library preparation was performed using the NEBNext^®^ Ultra DNA Library Prep Kit for Illumina. After PCR with the same barcoded primers, the purified amplicons were pooled in equimolar amounts and used as input for library preparation. The library was sequenced on the MiSeq platform using a MiSeq Reagent kit v2 following the 2x250-bp paired-end sequencing protocol.

With the aid of the second NGS-experiment we tried to find out potential influences of linked SNVs on the CpGs that are used by our technique. These NGS analyses were exclusively performed on the MiSeq platform. The resulting reads were selected according to the MiSeq quality criteria. The data were analysed in detail with a commercial software package CLC Genomics Workbench. As a quality control the data were filtered according to a Q-value of 30. The minimum coverage for a variant to be considered was 1000 reads, the forward/reverse balance was at least 0.3. However, in general there is a chance that some of the observed sequence variants reflect amplification errors e.g. early introduced in the amplification process. In our work exclusively variants have been considered, which exceeded the 1% limit and simultaneously revealed some impact on the methylation degree.

## Results

### Marker Selection

The genome wide screening using the Illumina HumanMethylation BeadChips 27k and 450k resulted in a total of 150 candidate sites with promising differential methylation properties regarding the relevant body fluids. These candidates were investigated in further validation studies by bisulfite sequencing and SNuPE tests. For each of the 150 candidate sites we designed an assay involving bisulfite converted DNA and 3–4 SNuPE-primers targeting the respective CpG. We verified the best candidate sites from the whole genome screenings by analyzing the samples used in the chip-experiments. The markers thus confirmed were analyzed on 5 further samples of each body fluid. Candidates were excluded if there were false negative signals in a target fluid sample or false positives in a sample of the remaining four body fluids. Finally we selected the best 9 marker loci containing one or two most discriminating CpGs for the ultimate validation using SNuPE assay. As described in Methods and Material the most CpGs were not identical with the ones found in the chip-analysis but were adjacent to them. Table 1 shows the genomic positions of the body fluid-specific CpGs that we used in msSNuPE assays and in addition the target CpGs that we originally selected from both Illumina chips 27k and 450k. The distances between the finally used CpGs and the chip-selected CpGs range from 0 to 288 bp (see genomic position in Table 1).

**Table 1:** Genomic description of the marker loci.

Marker	Target ID	Mean beta values					genomic position of Illumina Target CpG	genomic position of analytical CpG in msSNuPE	distance between analytical CpG and Illumina Target CpG
		Peripheral blood	Menstrual blood	Saliva	Vaginal fluid	Sperm			
Blut1	cg26285698	0,040483745	0,8105674	0,7953699	0,7893615	0,9202591	29,757,334	29,757,374 (r), 29,757,319 (f)	14/39
Blut2	cg03363565	0,55301355	0,3607591	0,04806649	0,04413896	0,046012115	474,528	474,528 (r), 474,468 (f)	0/59
Mens1	cg09696411	0,016833795	0,34692295	0,00865226	0,00714299	0,00714299	58,013,517	58,013,544 and 58,013,560	25/40
Spei1	cg21597595	0,02310832	0,024697213	0,427508	0,02327266	0,007995521	5,506,228	5,506,228 and 5,506,233	0/4
Spei2	cg15227982	0,28409295	0,1814097	0,69948255	0,100955	0,87509835	104,535,854	104,535,991	137
Vag1	cg14991487	0,049892885	0,6133649	0,046299455	0,7536326	0,0778048	176,987,404	176,987,319	85
Vag2	cg03874199	0,039484575	0,3200375	0,041379035	0,4448729	0,037350725	176,964,456	176,964,244	212
Sperm1	cg22407458	0,696400205	0,6162664	0,57111575	0,62528224	0,04203504	35,109,121	35,109,409	288
Sperm2	cg05656364	0,02123727	0,02316815	0,028549505	0,035558655	0,6478361	85,804,732	85,805,015	283

The markers Blut1, Spei2, Vag1, Vag2, Sperm1 and Sperm2 were selected from the study with the Human Illumina Methylation 27 BeadChip. The corresponding Target IDs could also be found on the 450k chip. The markers Blut2, Mens1 and Spei1 were exclusively selected out from the 450k chip data. Mean beta values were obtained from the 27 and 450 BeadChip analyses of 2 pooled samples of peripheral blood, menstrual blood, saliva, vaginal fluid and sperm respectively. The mean value is shown in the table. The last two columns describe the CpGs which were derived from the originally chip-data due to their higher discrimination power. The genomic positions of Illumina target CpGs and the analytical CpGs in msSNUPE refer to genome build 37/hg19.

Both for peripheral blood and semen fluid we developed a system consisting of two markers denoted here as reciprocal. Both systems have o ne marker being methylated in the target fluid and unmethylated in the remaining fluids (Blut2 for peripheral blood and Sperm2 for semen fluid) and a second one that is unmethylated in the target fluid but methylated in the others (Blut1 or rather Sperm1). This principle allows an even better analysis of complex mixtures, because admixtures of any other fluid produce a methylation signal. To further increase the reliability of the assay both Blut1 and Blut2 apply two different marker CpGs each, thus mutually confirming each other. We tested also the option of a diplex reaction this way. The marker loci for vaginal fluid (Vag1, Vag2), saliva (Spei1, Spei2) and menstrual blood (Mens1) are methylated in their target fluid and are hypomethylated in the others. This type of marker indicates the presence of the target fluid also in unknown mixtures with the other body fluids.

### Discrimination Power

**Fig 1:**
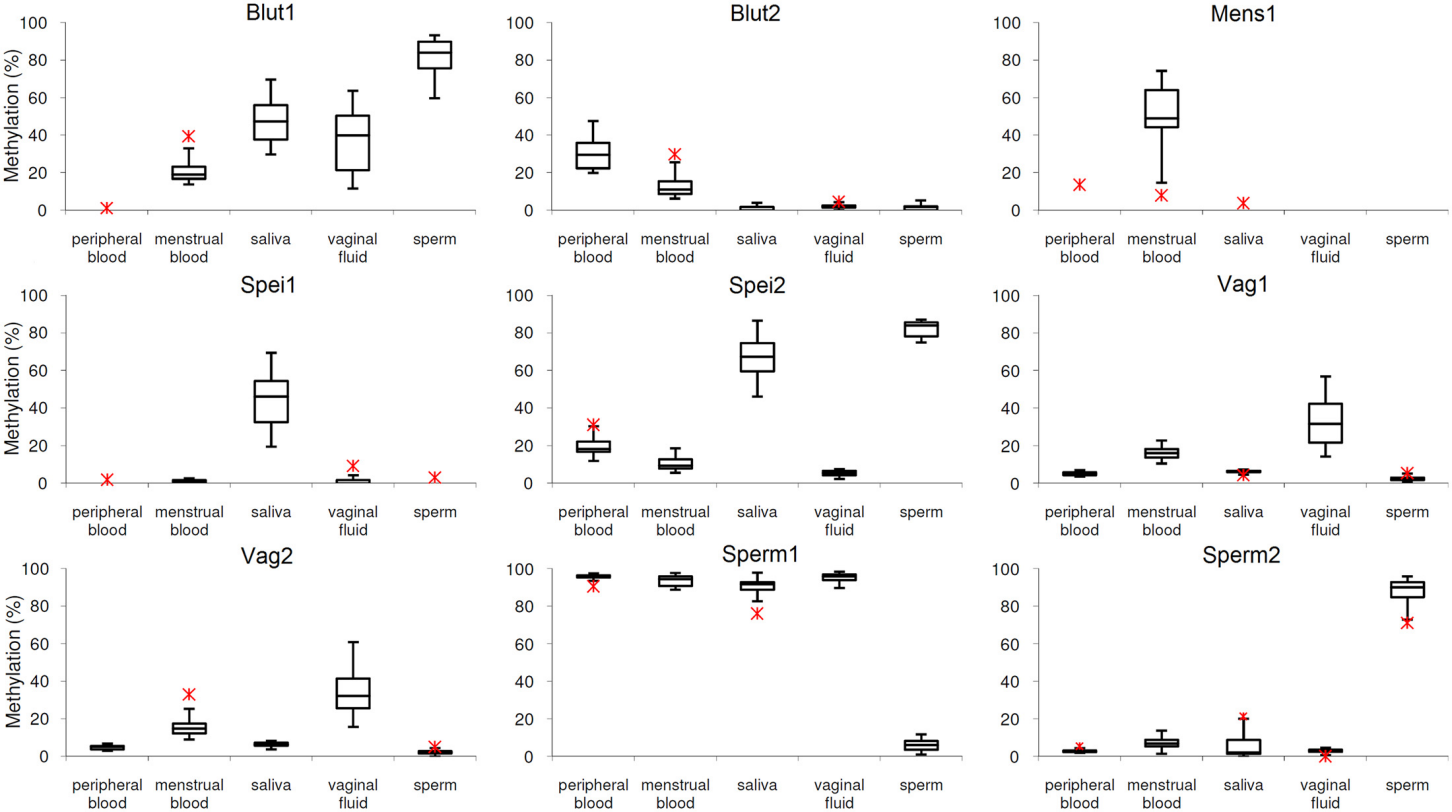
Boxplot diagrams showing the discriminant power of the 9 markers. They present the methylation rates for 20–22 samples per body fluid. Menstrual blood and vaginal fluid samples were collected by 22 healthy women (age 15 to 41 y, mean 30 y); the peripheral blood samples were taken from 9 healthy women (age 22–68 y, mean 38 y) and 11 healthy men (age 28–52 y, mean 39 y); sperm samples were given by 20 healthy men (age 20–42 y, mean 32 y); the saliva samples were collected from 12 healthy women (age 28–66 y, mean 35 y) and 8 healthy men (age 21–48 y, mean 39 y). Methylation values identified as outliers are marked with an asterisk. Only the lowest and highest outliers are shown.

The boxplot diagrams (Fig 1) demonstrate the capability of the system to identify one of the 5 body fluids frequently relevant for the investigative work in sexual assaults from a stain of unknown composition. These empirical data promise a high discrimination power since in all cases there is at least one marker for each kind of fluid, which shows non overlapping distribution of methylation rates compared to all other fluids. For all markers the methylation rates for the fluid in question are different from methylation rates of the other fluids (p < 0.0001 for all comparisons, nonparametric Dunnett). The detection limit of the different assays varies between 100pg and 200pg DNA. DNA content above this level generates stable and reproducible values in all markers. Provided enough material is available (1.3 ng DNA, 200 cells, bloodspot 0.5 mm^2^) each marker can be simultaneously applied. Each of the body fluids can be safely identified this way. However, the usual problem is a decision between two fluids and sometimes with much less material available. For example, the most frequently asked question is- do we have peripheral blood or menstrual blood? Even extreme values from peripheral blood and menstrual blood allow an unequivocal answer using Blut1, Blut2 and Mens1. The most efficient procedure to solve such problems is a stepwise analysis beginning with Mens1. In the same way the combined usage of Mens1, Vag1 and Vag2 clearly differentiates even in extreme values between vaginal fluid and menstrual blood. The remaining identification problems can be solved by using only two markers or even one specific for the interesting target fluid, because there is no overlap with any other fluid.

The body fluids discussed in this work are the most relevant for the investigation of sexual assaults but nevertheless false positive results might be obtained by any cross reaction with other body fluids or tissue. To exclude such events, we studied our 9 markers with the DNA of urine, seminal fluid of vasectomized men and skin. No conflicting results were observed (data not shown).

### Simulation of Forensic Conditions

In order to test the stability of the methylation degree under the exogenous impacts usually encountered at a crime scene, we performed a series of simulation experiments choosing standardized conditions [19]. The largest changes of the methylation signals were caused by humidity. Even after six months outside storage the system appears to be stable. Dry material remained unchanged in all markers and all body fluids over the whole time. The results are shown in Table 2.

**Table 2:** Simulation of forensic conditions.

	peripheral blood			menstrual blood			saliva			sperm			vaginal fluid		
	dry	humid	outdoor	dry	humid	outdoor	dry	humid	outdoor	dry	humid	outdoor	dry	humid	outdoor
Blut1	---	---	---	increasing with time			---	---	---	---	---	↓ 2, 6	---	↓ 2, 6	---
Blut2	---	↑ 6	---	---	---	---	---	---	---	---	---	---	---	---	---
Mens1	---	---	---	---	0↓ 6	---	---	↑ 2	---	---	---	---	---	---	---
Spei1	---	---	---	---	---	---	---	0↓ 3,6	---	---	---	---	---	---	---
Spei2	---	↑ 3	↑ 3	---	---	---	---	n.d. 3,6	0↓ 3	---	---	---	---	---	---
Vag1	---	---	---	---	↑ 6	↑ 6	---	---	---	---	---	---	---	↑ 2,3,6	---
Vag2	---	---	---	---	0↓ 3,6	---	---	---	---	---	---	---	---	↑ 2, nd 6	---
Sperm1	---	↓ 3	↓ 3	---	---	---	---	---	---	---	↑ 3, 6	---	---	---	---
Sperm2	---	↑ 3, 6	↑ 1,3,6	---	↑ 3	↑ 6	---	↑ 6	---	---	---	↓ 2,3,6	---	↑ 6	---

The results of the forensic simulation study performed with all markers on each body fluid are shown. The body fluid stains were exposed to three types of environmental influences (dry, humid and outside) up to six months. The initial analysis was done immediately after sample taking. Subsequently, the methylation values of the samples were determined after 1, 2, 3 and 6 months of storage. A stroke means stable during the whole period of storage. The arrows show whether the methylation signal decreased or increased after the elapsed time given in months, n.d. means not detected.

### Mixture Analysis

**Fig 2:**
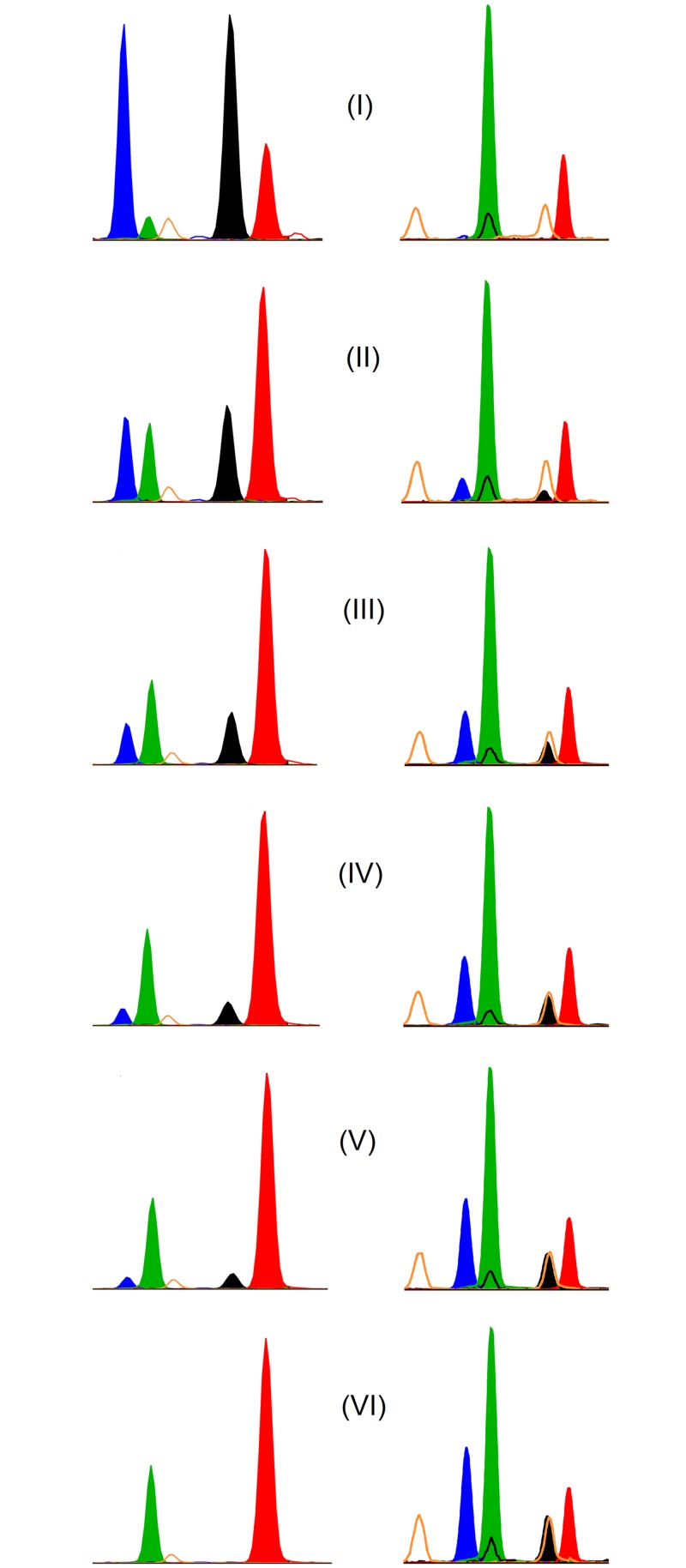
Results of mixture experiments for both peripheral blood markers. The specific reaction of Blut1 (left) and Blut2 (right) with blood DNA in a mixture with DNA of the remaining 4 fluids is shown. Both markers are diplex assays. 100% mixture + 0% blood (I), 80% mixture + 20% blood (II), 60% mixture + 40% blood (III), 40% mixture + 60% blood (IV), 20% mixture + 80% blood (V), 0% mixture + 100% blood (VI). Blut1 is umethylated in pure peripheral blood (green and red signal). Blut 2 is methylated in blood (blue and black signal).

To scrutinize any potential interference between the target fluid and other body fluids, we performed a mixture experiment. As an example Fig 2 shows the results for peripheral blood mixed up with different percentages of the remaining fluids. Slight effects were observed with Blut1-f in venous blood and the remaining four body fluids, as well as in vaginal fluid for both markers Vag1 and Vag2, Spei2 in saliva in a mixture with the other fluids. A remarkable deviation from the theoretical values was observed with Sperm2–20% higher than expected (S3 Fig).

### Extended Validation Study

In principle we selected most of the analytical CpGs according to their discrimination power being highly methylated in the target fluid and unmethylated in the others but as a matter of fact there are two groups of influences potentially making the distinctions less clear: Most body fluids are composed of different cell types and in some of them one of the other fluids naturally occurs as for menstrual blood or vaginal fluid. Furthermore, the degree of methylation is a quantitative factor potentially influenced by several biological parameters. As a consequence, an extended study revealing the character of the distribution of the methylation values appeared to be necessary. For that purpose we determined the methylation values of each marker in its target fluid in samples of menstrual blood (n = 96), venous blood (n = 100), vaginal fluid (n = 55), saliva (n = 100) and sperm (n = 91). The age of the probands represents the relevant life span in the forensic context (Table 3). As can be seen in S4 Fig the methylation values for all markers approximately show a normal distribution except for Sperm1.

**Table 3:** Numerical description of the extended validation.

Marker	Number of analyzed samples	Age variation of probands	Mean age of probands	Median of methylation values (%)	Mean value of Methylation (%)	Standard deviation Methylation (%)	Variation range Methylation (%)	Number of upper spikes	Number of lower spikes
Blut1-f	96	18–68	28	0	0,3	0,7	0–4,0	0	0
Blut1-r	96	18–68	28	0	1,3	1,0	0–4,2	0	0
Blut2-f	98	18–68	28	34,2	34,8	6,7	19,5–50,4	0	0
Blut2-r	98	18–68	28	35,0	35,3	7,6	18,9–52,7	0	0
Mens1	96	15–45	32	48,6	48,0	22,0	3,8–84,7	0	0
Spei1	99	1–67	27	53,7	51,5	18,8	5,8–89,4	0	1
Spei2	95	1–67	27	72,3	69,6	15,7	18,9–97,2	0	1
Vag1	55	21–70	35	39,7	38,9	16,4	14,3–92,0	3	0
Vag2	55	21–70	35	39,2	39,8	16,4	13,8–87,1	1	0
Sperm1	88	20–51	37	6,4	8,0	12,9	0–53,4	9	0
Sperm2	91	20–51	37	93,0	88,3	15,0	25,4–100	0	8

Results of extended validation study. The table shows the data of methylation values of the markers exclusively in their target fluids. See also S4 Fig.

### Quantifying the Methylation with Different Methods

**Fig 3:**
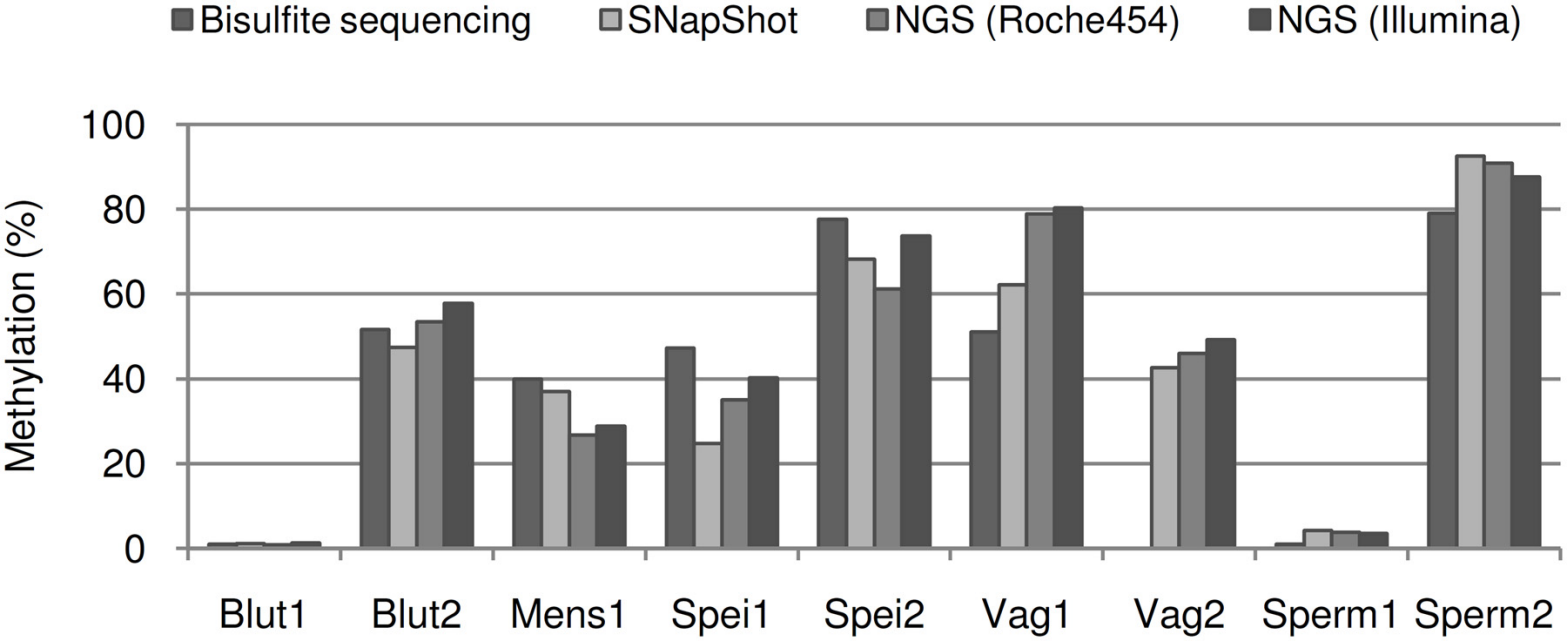
Comparison of the different methylation quantifying methods. Each marker was analyzed in one sample of the target fluid by bisulfite sequencing, SNaPshot reaction, Roche 454 and Illumina MiSeq NGS respectively.

Due to the crucial significance of the forensic body fluid analysis we tried to estimate how far the obtained SNuPE results deviate from the absolute methylation values. NGS-analyses might well reflect the biological reality at a CpG group. Therefore, we compared the bisulfite sequencing method, 454-sequencing, Illumina MiSeq-sequencing and the SNuPE-analysis (Fig 3). According to this experiment the SNuPE- results correspond to the NGS data very well.

### The Marker Loci and Tumor Effects

It has long been known that tumors may modify the methylation pattern of an affected person. However, this capability is probably confined to specific cells or tissues due to the cell specificity of the methylation [21]. Thus, one may assume that only the tumor affected tissue or celltype shows—if at all—such an altered methylation pattern. The tumor seems to pursue a strategy when changing the methylation of a genome: The 5' and 3' adjacent areas of a gene are very often hypermethylated, whereas the gene body is hypomethylated [22]. Tumor suppressor genes are frequent targets of these events. Because of the tissue/cell specificity of the methylation we investigated the following body fluids which might be functionally or locally linked to the respective tumor: venous blood/CLL, CML, MPS, Beta-cell lymphoma, colorectal carcinoma, breast cancer; vaginal fluid/cervixcarcinoma, ovarian carcinoma, breast cancer; saliva/esophageal carcinoma; seminal fluid/testis carcinoma.

**Fig 4:**
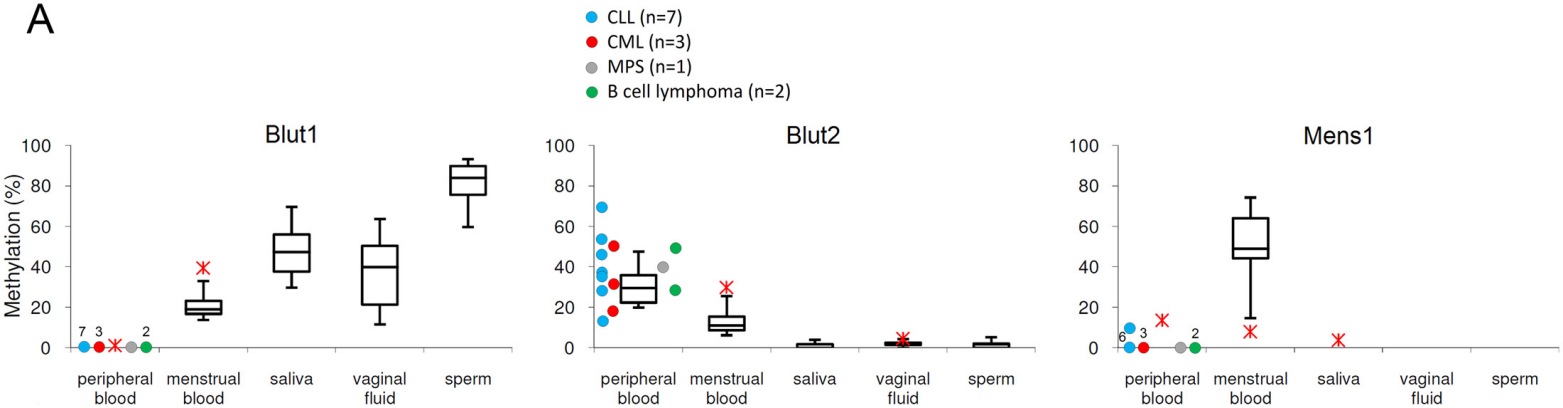
Methylation values in tumor related body fluids. **(A) Venous blood of CLL, CML, MPS and B-cell lymphoma patients.** The effect of CLL (chronic lymphocytic leukemia), CML (chronic myelocytic leukemia), MPS (myeloproliferative syndrome) and B-cell lymphoma was tested in venous blood of 13 patients. The obtained methylation values are shown as circles beside the boxplot diagrams from Fig 1. Each of the three markers relevant for venous and menstrual blood detection produced methylation signals in the range of normal controls except for one CLL and one CML sample with Blut2 in peripheral blood here revealing some overlap with the menstrual blood distribution. The remaining Blut2-values rather increased the discrimination power. Each of the 13 samples showed a zero signal with Blut1. One CLL sample showed a slightly elevated signal with Mens1.**(B)Venous blood of breast cancer and colorectal carcinoma patients.** The effect of breast cancer and colorectal carcinoma was tested in venous blood of 12 patients. The obtained methylation values are shown as rhombs beside the boxplot diagrams from Fig 1. All of the three markers relevant for venous and menstrual blood detection showed methylation values in the normal range except for one blood sample of a patient with breast cancer and one blood sample of a patient with colorectal carcinoma (HNPCC hereditary nonpolyposis colorectal cancer). Nevertheless, they still discriminate between the bodyfluids although overlapping with the menstrual blood distribution. Each of the 12 samples showed a zero signal with Blut1 and Mens1. **(C) Vaginal fluid of endometrial cancer, cervix carcinoma, ovarian carcinoma and endometriosis patients.** The influence of endometrial carcinoma, cervix carcinoma, ovarian carcinoma and endometriosis was analysed in 6 vaginal fluid samples. The obtained methylation values are shown as triangles beside the boxplot diagrams from Fig 1. Both Cervix carcinoma samples showed with both vaginal fluid specific markers methylation values at the lower part of the distribution thus overlapping with the menstrual blood distribution. The remaining values did not compromise a decision for vaginal fluid. **(D) Saliva of an esophageal carcinoma patient.** In one case of esophageal carcinoma we analysed the methylation with both saliva markers in saliva. The obtained methylation value is shown as a square beside the boxplot diagrams from Fig 1. The Spei1 value was lower than normal; the Spei2 value was in the higher normal range thereby maintaining the discriminant power. **(E) Sperm of testicular carcinoma patients.** We studied the potential impact of testicular carcinoma in the sperm of two affected patients.The obtained methylation values are shown as rectangles beside the boxplot diagrams from Fig 1. The signals of Sperm1 were slightly above the normal controls, whereas the Sperm2 values corresponded to normal controls.

As shown in Table 4 we observed several remarkable effects of the tumors, even if the discrimination properties in most cases are maintained (Fig 4A–4E). The samples from patients suffering from the same tumor type do not always react the same way. In most cases it is only a part of the samples that reveal aberrant methylation compared to normal controls. The important question is: Does the tumor distort the results and produce false positives or negatives? As mentioned above, there are two ways to use this set of markers. The first one is to apply all markers in order to characterize a completely unknown biological trace, the second one is to safely identify one body fluid or to make a decision between two of them. Considering the first situation one would obtain a confusing result in case of a blood sample originating from a CLL patient, because Mens1 recognizes menstrual blood, Spei1 and Spei2 saliva, Vag1 and Vag2 vaginal fluid, Sperm2 the presence of seminal fluid, but Blut2 recognizes peripheral blood and also Blut1 correctly excludes the presence of other fluids. However, such a result would induce the right conclusion that the sample is peripheral blood—most probably from a CLL patient. A similar situation is observed for samples with venous blood from CML, MPS and beta-leukemia patients: Vag1 and Vag2, Sperm1 and Sperm2 present conflicting results but the markers for the target fluid—Blut1 and Blut2 —recognize pure blood. The identification of one or two body fluids using only the specific markers appears to be undisturbed, except for vaginal fluid of cervix carcinoma patients, where the methylation significantly decreases in both markers thus potentially resulting in a false negative result (see Fig 4C).

**Table 4:** Effect of different carcinomas.

Type of carcinoma	Blut1	Blut2	Mens1	Spei1	Spei2	Vag1	Vag2	Sperm1	Sperm2
CLL	---	---	1/7 (10↑)	2/7 (3–6↑)	4/7 (22–57↑)	4/7 (7–38↑)	6/7 (19–54↑)	1/7 (40↓)	---
CML	---	---	---	---	---	1/3 (9↑)	1/3 (15↑)	1/3 (19↓)	2/3 (13–25↑)
MPS	---	---	---	---	---	---	1/3 (8↑)	1/3 (49↓)	---
B-cell lymphoma	---	---	---	---	---	1/2 (13↑)	1/2 (29↑)	---	---
cervixcarcinoma	---	---	---	1/2 (5↑)	2/2 (12–62↑)	2/2 (13–17↓)	1/2 (12↓)	1/2 (23↓)	---
ovarian carcinoma	---	---	---	1/2 (6↑)	---	---	---	---	---
breast carcinoma	---	1/9 (20↓)	---	---	---	---	---	---	---
colorectal carcinoma	---	1/3 (19↓)	---	---	---	---	---	---	---

Only methylation loci affected by one or more carcinomas are shown. CLL (chronic lymphatic leukaemia), CML (chronic myelocytic leukaemia), MPS (myeloproliferative syndrome), B-cell lymphoma, breast carcinoma and colorectal carcinoma were analyzed in peripheral blood of the patients; Cervixcarcinoma and ovarian carcinoma were analyzed in vaginal fluid of the patients. The upper vertical line: type of the affected marker loci. The tumor driven effect is described by the number of affected samples out of the number of analysed samples (n/m) and the degree/ direction of effect (↑↓). The number means the absolute difference to the average percentage of methylation. A stroke means no effect on methylation degree.

### Genetic Influences

We performed our NGS-pool-experiment processing samples from 50 individuals, in order to elucidate genetic factors that influence or control the methylation of closely linked CpGs. As shown above, the methylation values determined by the SNuPE assay, the Roche 454 and MiSeq sequencing tightly correspond to each other. So, one can conclude that both the SNuPE assay and the MiSeq sequencing generate a reliable measurement. After the quality control and selection (see materials and methods) 230383 reads left. The coverage for the individual marker loci ranged from 25598 (Spei2) to 50928 (Sperm1).

**Fig 5:**
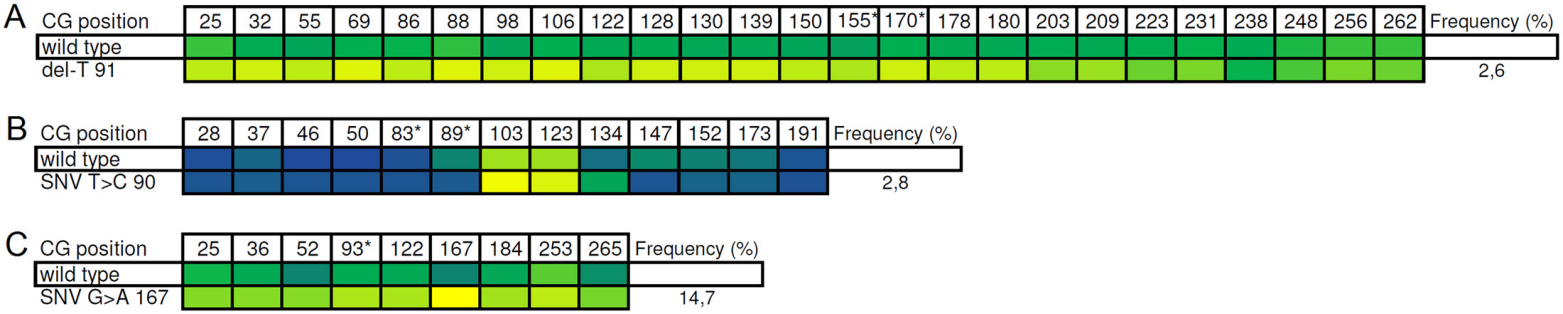
Marker loci influenced by single nucleotide variants (SNV). Only sequence variants with influence on the methylation of a marker locus are shown. Left vertical column: Type and position of variant. The upper horizontal line: position (number) of the CpG. The second line: normal methylation state without influence. Colours reflect the methylation degree of the CpG, increasing from yellow (0% methylated) to blue (100% methylated). CG positions marked with a star represent the analytical CGs where the primer extension takes place. Mens1: (A), Spei1: (B), Spei2: (C). Frequency of reads containing the SNV is 2,6% (A), 2,8% (B) and 14,7% (C).

**Fig 6:**
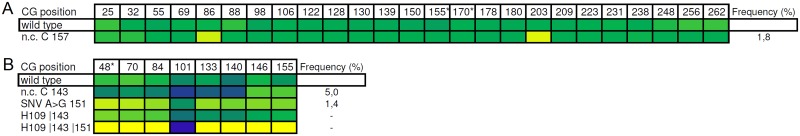
Marker loci influenced by not converted cytosines (n.c. C). Only sequence variants with influence on the methylation of a marker locus are shown. Left vertical column: Type and position of variant (H means haplotype of several variants). The upper horizontal line: position (number) of the CpG. The second line: normal methylation state without influence. Colours reflect the methylation degree of the CpG, increasing from yellow to blue. CG positions marked with a star represent the analytical CGs where the primer extension takes place. Mens1: (A), Vag2: (B). Frequency of reads containing the not converted cytosine is 1,8% for C157 (A) and 5,0% for C143 (B).

We observed three types of variants revealing a more or less pronounced cis-effect on the methylation of nearby CpGs: Small deletions and insertions, all of them as part of a homopolymer stretch, several types of nucleotide substitutions (Fig 5) and an unknown type that we called “nonconverted C” (Fig 6). A detailed description of the marker loci and their genomic surrounding is shown in S3 Table.

## Discussion

In this paper we describe a method that allows the detection and semiquantitative determination of venous blood, menstrual blood, saliva, vaginal fluid and sperm from forensic material. Because for venous blood and sperm the analysis of mixtures appears to be especially important, we developed for both body fluids complementary marker systems: One marker is methylated exclusively in the target fluid, the second marker is methylated in the remaining fluids but not in the target fluid. Thus, the first marker identifies the target even in complex mixtures; the second marker announces the presence of any other fluid discussed in this context. For vaginal fluid and saliva we developed a system with two markers located on different chromosome positions or different chromosomes respectively, both markers indicating the target fluid when being methylated. For menstrual blood one strong discriminating marker was developed in this work. A second one is generated but not described in this paper.

For all but one body fluid there is at least one marker showing an empirical distribution of methylation percentages which does not overlap with the values measured for the other fluids. At least the combination of two markers allows the complete separation of the fluids (Vaginal fluid and menstrual blood). The estimated rates of correct predictions of body fluids, based on this data, are therefore always 100%, with precision that is limited by the size of the validation samples.

Convincing studies have shown that the methylation pattern is age-dependent [23]. Therefore, in principle our marker loci could be affected by the age of the person who originated the trace. Testing the results of our validation study we observed a weak correlation between age and the methylation degree only in both vaginal markers Vag1 and Vag2 in vaginal fluid (R = 0,43 and 0,42). But this correlation does not compromise the discrimination power of the markers. The correlation of the remaining markers was not significant (data not shown).

For any forensic application the stability of the analytical substrate is of crucial importance. In principal exogenous impacts may affect at the analytical CpG or the methylation state or at the whole PCR-template. The 5-Methyl Cytosine tends to spontaneously deaminate in the natural environment of the cell. Also bacteria or funghi may have this effect. If so, it would compromise the marker loci which are methylated in the target body fluid. The chemical stability of the template DNA itself corresponds to any other type of dsDNA. In our forensic simulation experiments we observe preferentially in the samples which were exposed to a humid environment some influence on the methylation signals over the time but it does not distort the correctness of the identification of the body fluids.

Evidently, the methylation degree of a CpG is not always genetically controlled with 0% or 100% methylation as for regions of parental imprinting or inactivation of the X-chromosome. In many gene loci it is somewhere in between—even in identical cell lines [17]. Nevertheless, our measurements form approximately a normal distribution as shown in Table 3 and S4 Fig. The collection of menstrual blood samples comprises day 1–5. Most samples are from day 2, which is perceived by most women as the day of strongest bleeding, whereas day 5 is often disregarded. Twelve methylation values of the menstrual blood entity were comparatively low. Most probably this has been caused by inappropriate sample taking. The Mens1 marker is directed to endometrium tissue, which is not always safely absorbed by the tampons which sometimes were used for collecting the material. The frequently requested discrimination between venous blood, menstrual blood or a mixture of both can be clearly achieved by using Blut 1-f, 2-f and Mens1 even with extreme methylation values from the edge of the distribution: Blut1-f unmethylated and Blut2-f methylated means the presence of pure venous blood, Blut1-f and Blut 2-f methylated means the presence of menstrual blood, which is confirmed by methylated Mens1.

As shown in Fig 2 the detection of venous blood is also possible from a mixture containing one or more of the remaining body fluids with more than 20% blood. Mens1 is exclusively methylated in menstrual blood and unmethylated in the other body fluids. Spei1 shows no overlap with any other body fluid. Spei1 identifies saliva in any mixture containing more than 20% saliva. The same applies to Vag2. Using the Mens1 marker facilitates an unequivocal decision in case of such potentially ambiguous results. In case of seminal fluid, it is very often necessary to analyze a more or less complex mixture. Therefore we developed the reciprocal marker system mentioned above. There is no overlap between the methylated Sperm2 and any other marker signal. Sperm2 identifies seminal fluid in an arbitrary mixture containing more than 20% of the target fluid.

The methylation degree of all CpGs in a tumor cell is decreased to 40–60% of the normal cell [22]. In this study we tested the potential effect of some relatively common tumors on the methylation of our individual marker loci. As a matter of fact, such a study leaves the question unanswered, whether any rare tumor may change the methylation pattern of an affected. Nevertheless, the evaluation of these experiments significantly increases the overall reliability of the method.

Taken as a whole, the different tumors studied in our work show a lot of effects on the various methylation loci discussed here, but only the interaction between the cervix carcinoma on one hand and Vag1 and Vag2 on the other might compromise the quality of the corresponding test—both markers show decreased methylation values compared to the normal controls thus overlapping with the menstrual blood signals. In part, the observed modifications of the methylation correspond to mechanisms known from tumor studies: In Mens1 the analytical CpG is part of a CpG island situated 5' to the gene SLC26A10—the methylation is increased in a CLL-patient. The diagnostic Vag2 CpG is located 75 bp 5' to HOXD12 as part of a CpG island. The increased methylation rate in patients with CLL, MPS and B-cell lymphoma is in accordance with the tumor strategy as well. The decreased methylation of Sperm1 in one blood sample of CLL, CML and MPS patients respectively and in the vaginal fluid of one patient may reflect the described tumor induced hypomethylation of the gene bodies. Sperm1 maps to the 3'end of the last exon of a TCP11 gene. The same applies to the increased methylation of Sperm2 in 2 out of 3 venous blood samples of CML patients. The Sperm2-locus is part of a CpG island within the 3’-untranslated region of VAMP8. The remaining tumor driven modifications observed in our experiment cannot be explained by the known mechanisms. As a consequence of these results one can conclude that even the body fluids of tumor affected individuals can be identified with a high degree of certainty. An exception might be the identification of vaginal fluid by means of Vag1 and Vag2.

The variance of the methylation values may be influenced by sequence variants [15]. To explore such interactions we carried out our NGS experiment. Against the background of our specific technology the effect of sequence variants could take place on different levels: There may be cis-regulatory mechanisms that account for ASM and there may be sequence variants within the primer binding sequence or even in the diagnostic CpG thus impairing the SNuPE reaction. One aspect in selecting appropriate marker loci from our two genome wide methylation studies was to minimize the presence and density of SNPs. However, private, so far undetected sequence variants will usually be found when sequencing intergenic regions. Our NGS study revealed in the following amplicons a cis-effect of sequence variants to closely linked methylation loci. Although being part of a T-stretch, we consider del-91 in the Mens1 locus a real mutation rather than a sequencing artifact due to the remarkable effect on the methylation of Mens1. It decreases the methylation of Mens1 thereby decreasing the sensitivity of the test system. We observed it in 2.6% of the Mens1 reads. The sequence variant T>C 38 (4% of the reads) increases the methylation of Spei1 in the diagnostic CpGs 83 and 89. Because Spei1 is methylated in the target fluid, the variants would not impair the test. The sequence variant G>A167 (14.7% of the reads) decreases the methylation of the analytical CpG in Spei2. We found an additional factor influencing the methylation of CpGs: Some amplicons contain Cs being not part of a CpG, which are not converted to T by the bisulfite reaction. But Cs outside CpGs should be unmethylated. A few of these Cs present the adjacent CpGs at a higher methylation rate than normal at this locus. Thus, this effect might be just a consequence of an incomplete bisulfite reaction. However, the remaining Cs outside CpG react in the expected way to T. One might speculate that this effect is produced by hydroxymethylation of these nonconverted Cs, which is known to regulate the methylation of neighboring CpGs [24]. On the other hand we observed in the Vag2 –locus non converted/non CpG positions (C143, C151) and the SNV A>T 109 revealing a slight to moderate effect on the methylation. Though, combined to one haplotype (SNV109, C143, C151) they produce a distinct change of the methylation pattern. Also in this case a sequencing artifact or any problem with the bisulfite reaction appears unlikely. Our NGS experiments show that sequence variants significantly contribute to the variance of the various distributions. However, at least within this cohort of 50 individuals we do not observe an example of completely impairing the test by a sequence variant. In no case we observed a sequence variant that destroys the structure of an analytical CpG itself. There is also no SNP described in literature whose rare allele would impair one of the diagnostic CpGs.

During the last five years several approaches predominantly based on mRNA studies for identifying body fluids from forensic material have been published [25–29]. In most samples the RNA-marker set allowed to identify the target fluid. Each of the studies comprised 6–10 samples. Numerous cross reactions between the different fluids are mentioned. The protocol uses a pattern of RNA signals, thus the analysis of a mixture appears to be extremely difficult. Due to its higher stability microRNA (18–20bp) has become an alternative molecule species which might replace the much more sensitive mRNA [7, 8]. Hansen et al discuss the combined use of 179 marker microRNAs forming a tissue specific expression pattern. Also this approach does not allow the detection of the target fluid in a mixture of others. Frumkin et al [10] applied methylation sensitive restriction enzymes (MSRE-PCR) to identify venous blood, saliva, seminal fluid, vaginal fluid, urine and skin. They used 15 differentially methylated regions (tDMRs) generating a characteristic fragment pattern. The system allowed the correct detection of seminal fluid even in a mixture with saliva. As a consequence, a five marker assay was validated analyzing 135 samples [30]. Lee et al [9] selected 5 differentially methylated candidate loci from a literature search. In a subsequent study [31] they analyzed 6 female and 10 male samples for venous blood, menstrual blood, saliva, semen fluid and vaginal fluid using these methylation loci. Except for semen, these markers are also unable to detect the target in a mixture. In a more recent work the same group describes eight body-fluid specific methylation markers (3 for semen, 2 for peripheral blood, 2 for vaginal fluid and 1 for saliva). They were analyzed on 100 samples using multiplex methylation SNaPshot. Obviously, this set does not contain a marker specific for menstrual blood. The authors also used the Illumina HumanMethylation450 BeadChip array [11]. Nevertheless, there is no overlap between the target IDs of the selected CpG sites in the Korean and our study.

Regarding the fact that also any RNA expression depends on the genetic, metabolic and the pathological situation of the cell we believe that the procedure presented in this paper is the most reliable forensic body fluid tool, because the more important potential influences have been tested in this study. In addition, our protocol appears to be superior compared to the previously published procedures due to its high potential of identifying the individual body fluids from mixtures.

A multiplexed NGS approach of the marker loci might be a logical and useful extension of the system presented in this paper, because it would allow the simultaneous analysis of effective sequence variants and several discriminating loci as used in our procedure.

## Supporting Information

S1 Fig (PDF)

S2 Fig (PDF)

S3 Fig (PDF)

S4 Fig (PDF)

S1 Table (PDF)

S2 Table (PDF)

S3 Table (PDF)
