# Correction: Methylation Markers for the Identification of Body Fluids and Tissues from Forensic Trace Evidence

**DOI:** 10.1371/journal.pone.0156472

**Published:** 2016-05-24

**Authors:** Sophia Forat, Bruno Huettel, Richard Reinhardt, Rolf Fimmers, Gerhard Haidl, Dominik Denschlag, Klaus Olek

The image for Fig 4 is incorrect. Please see the correct Fig 4 here.

**Fig 4:**
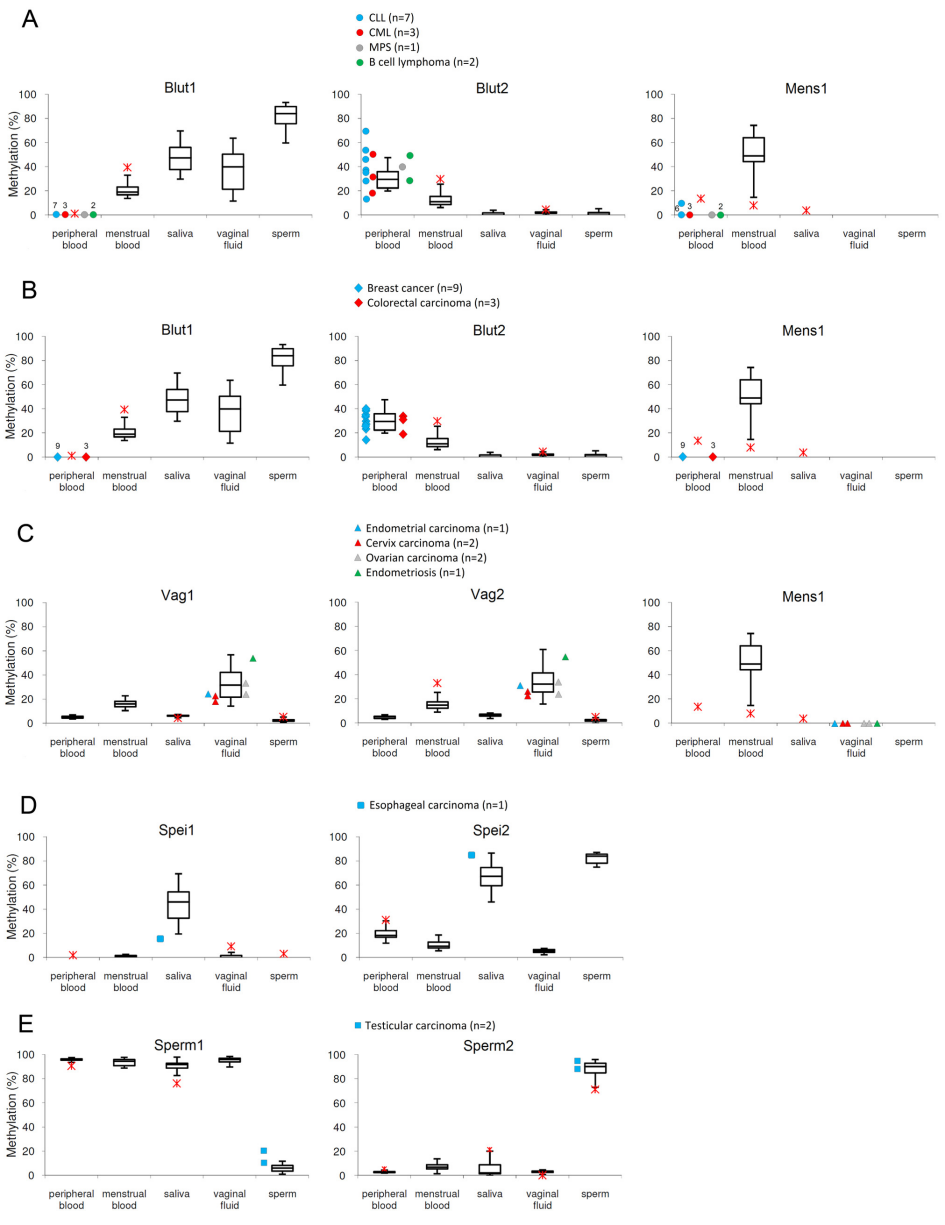
Methylation values in tumor related body fluids. **(A) Venous blood of CLL, CML, MPS and B-cell lymphoma patients.** The effect of CLL (chronic lymphocytic leukemia), CML (chronic myelocytic leukemia), MPS (myeloproliferative syndrome) and B-cell lymphoma was tested in venous blood of 13 patients. The obtained methylation values are shown as circles beside the boxplot diagrams from Fig 1. Each of the three markers relevant for venous and menstrual blood detection produced methylation signals in the range of normal controls except for one CLL and one CML sample with Blut2 in peripheral blood here revealing some overlap with the menstrual blood distribution. The remaining Blut2-values rather increased the discrimination power. Each of the 13 samples showed a zero signal with Blut1. One CLL sample showed a slightly elevated signal with Mens1. **(B)Venous blood of breast cancer and colorectal carcinoma patients.** The effect of breast cancer and colorectal carcinoma was tested in venous blood of 12 patients. The obtained methylation values are shown as rhombs beside the boxplot diagrams from Fig 1. All of the three markers relevant for venous and menstrual blood detection showed methylation values in the normal range except for one blood sample of a patient with breast cancer and one blood sample of a patient with colorectal carcinoma (HNPCC hereditary nonpolyposis colorectal cancer). Nevertheless, they still discriminate between the bodyfluids although overlapping with the menstrual blood distribution. Each of the 12 samples showed a zero signal with Blut1 and Mens1. **(C) Vaginal fluid of endometrial cancer, cervix carcinoma, ovarian carcinoma and endometriosis patients.** The influence of endometrial carcinoma, cervix carcinoma, ovarian carcinoma and endometriosis was analysed in 6 vaginal fluid samples. The obtained methylation values are shown as triangles beside the boxplot diagrams from Fig 1. Both Cervix carcinoma samples showed with both vaginal fluid specific markers methylation values at the lower part of the distribution thus overlapping with the menstrual blood distribution. The remaining values did not compromise a decision for vaginal fluid. **(D) Saliva of an esophageal carcinoma patient.** In one case of esophageal carcinoma we analysed the methylation with both saliva markers in saliva. The obtained methylation value is shown as a square beside the boxplot diagrams from Fig 1. The Spei1 value was lower than normal; the Spei2 value was in the higher normal range thereby maintaining the discriminant power. **(E) Sperm of testicular carcinoma patients.** We studied the potential impact of testicular carcinoma in the sperm of two affected patients. The obtained methylation values are shown as rectangles beside the boxplot diagrams from Fig 1. The signals of Sperm1 were slightly above the normal controls, whereas the Sperm2 values corresponded to normal controls.

## References

[1] Forat S, Huettel B, Reinhardt R, Fimmers R, Haidl G, Denschlag D, et al (2016) Methylation Markers for the Identification of Body Fluids and Tissues from Forensic Trace Evidence. PLoS ONE 11(2): e0147973 doi:10.1371/journal.pone.0147973 26829227 10.1371/journal.pone.0147973 PMC4734623

