# Supplementary material for: Methylation Markers for the Identification of Body Fluids and Tissues from Forensic Trace Evidence
Source: PLoS One. 2016 Feb 1;11(2):e0147973. doi: 10.1371/journal.pone.0147973 (PMC4734623; doi:10.1371/journal.pone.0147973)
Supplement: S1 Fig — (PDF) [file pone.0147973.s001.pdf]

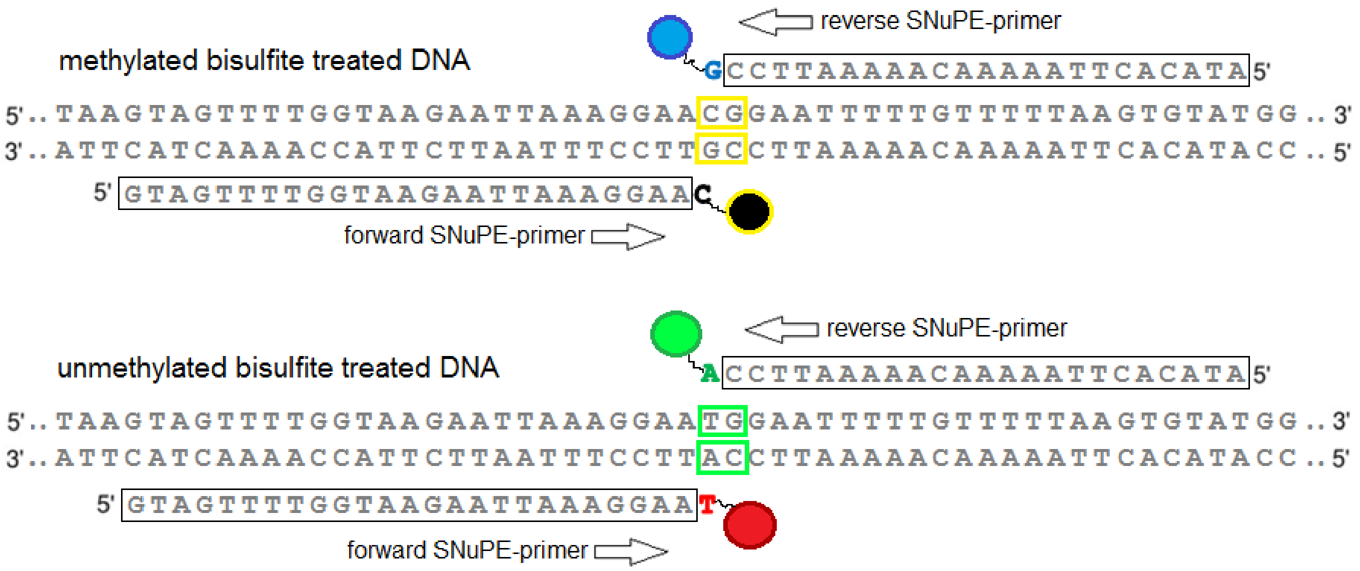

Fig. S1. Detection of DNA methylation using standard SNuPE-primer (both forward and reverse directions are shown). The primers anneal straight 5´ of the nucleotide to be detected and are extended on the 3´tail by one complementary fluorescent labeled didesoxynucleotide. To anneal on both sequence types - methylated and unmethylated - the target sequence of the primer may not contain further CpGs.
