# Supplementary material for: Methylation Markers for the Identification of Body Fluids and Tissues from Forensic Trace Evidence
Source: PLoS One. 2016 Feb 1;11(2):e0147973. doi: 10.1371/journal.pone.0147973 (PMC4734623; doi:10.1371/journal.pone.0147973)
Supplement: S2 Fig — (PDF) [file pone.0147973.s002.pdf]

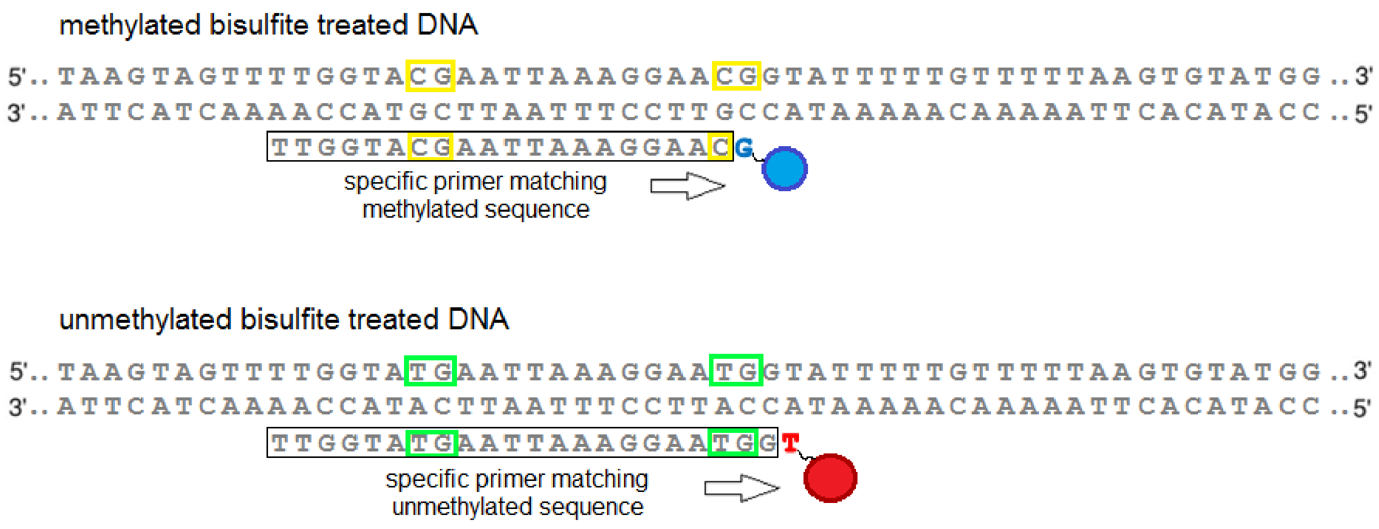

Fig. S2. Detection of DNA methylation using methylation specific SNuPE-primer (only forward detection is shown). The primers anneal 5´ of two different nucleotide types. The primer sequence covers several CpGs inside. This CpGs may be methylated or unmethylated. Thus, simultaneously are used one primer completely matching to the methylated sequence, the other one to unmethylated.
