# Supplementary material for: Methylation Markers for the Identification of Body Fluids and Tissues from Forensic Trace Evidence
Source: PLoS One. 2016 Feb 1;11(2):e0147973. doi: 10.1371/journal.pone.0147973 (PMC4734623; doi:10.1371/journal.pone.0147973)
Supplement: S3 Fig — (PDF) [file pone.0147973.s003.pdf]

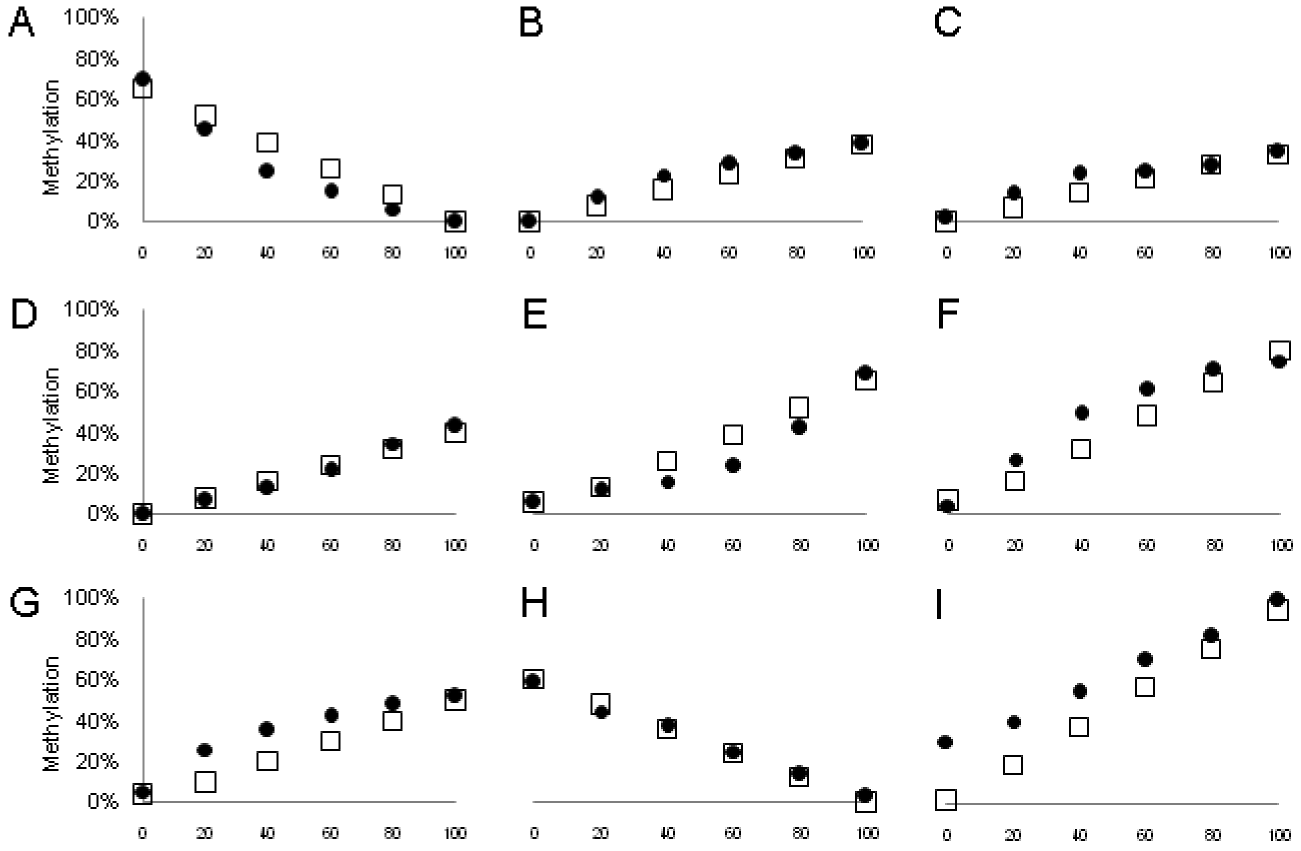

Fig. S3. Detection of the target fluid in mixtures containing 0, 20, 40, 60, 80 and 100 percent DNA of the specific fluid. The observed methylation value (●) and the expected one (□) of the mixtures are compared. For peripheral blood: (A) Blut1-f and (B) Blut2-f, for menstrual blood: (C) Mens1, for saliva: (D) Spei1 and (E) Spei2, for vaginal fluid: (F) Vag1 and (G) Vag2, for sperm: (H) Sperm1 and (I) Sperm2.
