# Supplementary material for: Methylation Markers for the Identification of Body Fluids and Tissues from Forensic Trace Evidence
Source: PLoS One. 2016 Feb 1;11(2):e0147973. doi: 10.1371/journal.pone.0147973 (PMC4734623; doi:10.1371/journal.pone.0147973)
Supplement: S4 Fig — (PDF) [file pone.0147973.s004.pdf]

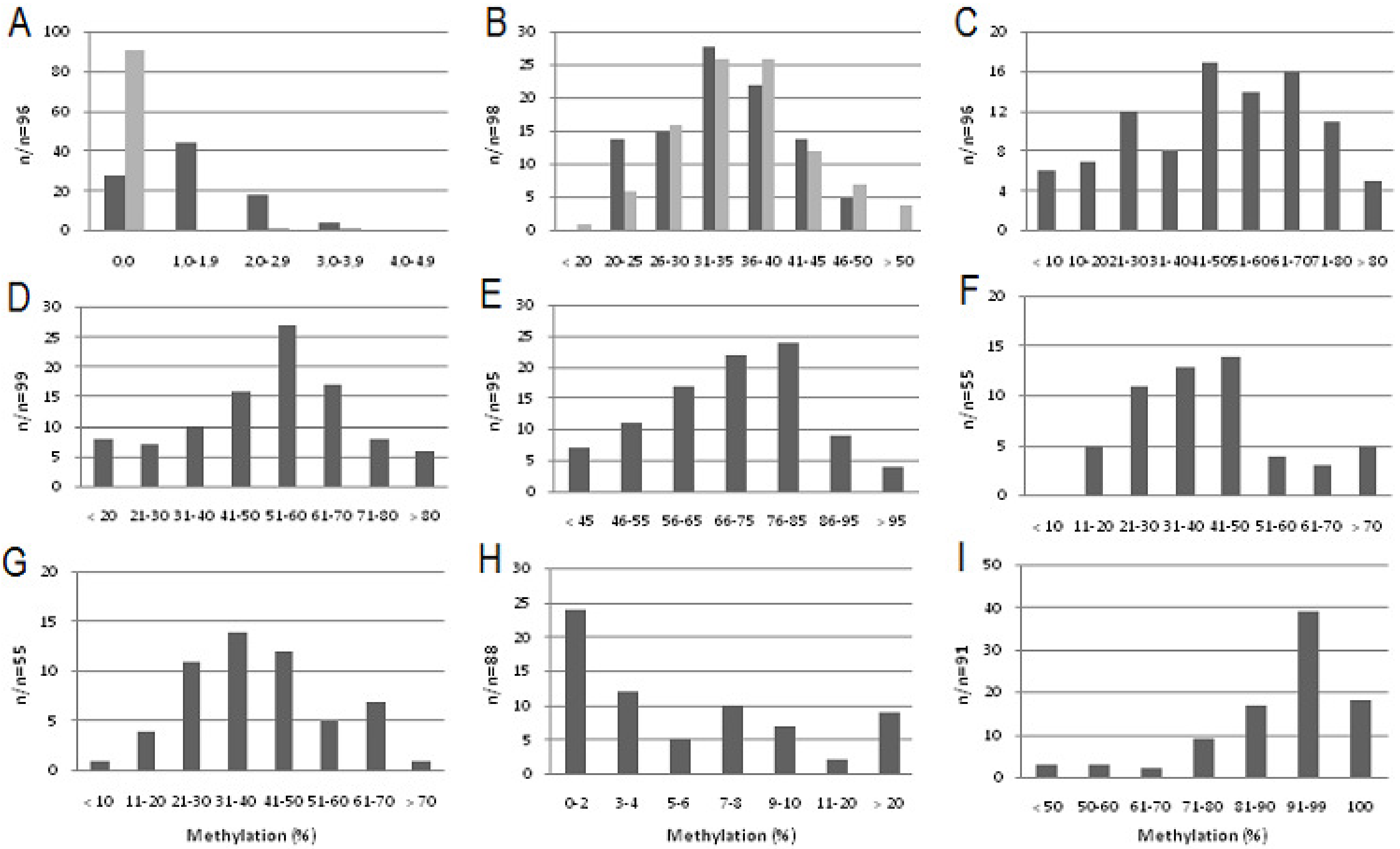

Fig. S4. Results of the extended validation study to determine methylation values of each marker in its target fluid. The methylation values are classified to individual groups on x-axis. The y-axis shows the number of the values. (A) Blut1-f and Blut1-r (B) Blut2-f and Blut2-r, (C) Mens1, (D) Spei1, (E) Spei2, (F) Vag1, (G) Vag2, (H) Sperm1 and (I) Sperm2.
