# Supplementary material for: Methylation Markers for the Identification of Body Fluids and Tissues from Forensic Trace Evidence
Source: PLoS One. 2016 Feb 1;11(2):e0147973. doi: 10.1371/journal.pone.0147973 (PMC4734623; doi:10.1371/journal.pone.0147973)
Supplement: S1 Table — (PDF) [file pone.0147973.s005.pdf]

Table S1. Bisulfite-specific PCR primers

Name	Forward primer (5´-3´)	Reverse primer (5´-3´)
Blut-1	TGG GTT GTT TTG GAA ATA AAT A	CCT CTA CAC CCC TCC TAA A
Blut-2	TAT AAT TTG TAT TAG GGA AAT GAT GA	TCC CTC ACA TTC CTT TTC C
Mens-1	GAT TAG GTT TAG GGA AGT TTT TAT	ACC CTC TAA AAC TTA TAC TCC C
Spei-1	CTA CAA AAA TAA ATA TAA ATA TAA AA	TTT TGG TGG TTT GGG GTT TA
Spei-2	ATT TCC CCC TTA ACA AAC AA	AAA AGG AAA GGT ATT TTG TAA GAG
Vag-1	GGT ATA TGG ATT TGG GTT TG	AAT TAC TAA AAA TAC CAC TAA AAA ACA T
Vag-2	ATT AAG AAG AGT TTT AGG GGA TAT	TTT CCC CCC CTT CAA AAT
Sperm-1	CAT ACA TAA AAC TTT TCT TCA AAC TAT	AAA GGT AAG GGT TAG AGT TTA G
Sperm-2	ACT AAA ATC TAA ACT AAA AAC TAC CC	ATA GTT TTG AGT TGT TTT GGT AGG TG

During bisulfite conversion, unmethylated cytosines are chemically converted to uracils, which will be amplified as thymidine. After bisulfite conversion the DNA strands are no longer complementary. PCR primers are specific for the first bisulfite converted DNA strand (Blut1, Blut2, Mens1, Vag1 and Vag2) or the second one (Spei1, Spei2, Sperm1 and Sperm2).
