# Supplementary material for: Methylation Markers for the Identification of Body Fluids and Tissues from Forensic Trace Evidence
Source: PLoS One. 2016 Feb 1;11(2):e0147973. doi: 10.1371/journal.pone.0147973 (PMC4734623; doi:10.1371/journal.pone.0147973)
Supplement: S2 Table — (PDF) [file pone.0147973.s006.pdf]

Table S2. Single base extension primers

Name	Forward SNuPE primer (5´-3´)	Reverse SNuPE primer (5´-3´)
Blut1-1f+2r	GGG GTA TAG TTG TTT AGT AAT AGG	CTA AAA AAT ACA AAC TTA AAA AAT ACA AAA C
Blut1-1f+2r	GTA GTT TTG GTA AGA ATT AAA GGA A	CCA TAT ACC CTA AAA TTC TAC CAC
Mens1-F3m	GGC GTT TGT TGT TGG TTC	---
Mens1-F3nm	GTG TTT GTT GTT GGT TTG G	---
Spei1-Fm	CTT TCA CAA CGC TCA CG	---
Spei1-Fnm	CTT CTT TCA CAA CAC TCA CAT	---
Spei2-3r	---	GAG TAG GTA TAT TGT TTT TTG TTT TTT T
Vag1-1r	---	TAA TTA AAA AAA AAA TCA TCA ACA TAA AC
Vag2-1f	ATA TTT GGG TAG AGT TAT TTT TTT GTT	---
Sperm1-4r	---	TTT TTT TTA TAT GTG AGG AAA GAG
Sperm2-3r	---	GTG GGG AGT TGG GTT TT

SNuPE primer sequences are specific for the first bisulfite converted DNA strand (Blut1, Blut2, Mens1, Vag1 and Vag2) or the second one (Spei1, Spei2, Sperm1 and Sperm2). In methylaton specific primers (Mens1 and Spei1) the discriminating bases are underlined.
