# Supplementary material for: Methylation Markers for the Identification of Body Fluids and Tissues from Forensic Trace Evidence
Source: PLoS One. 2016 Feb 1;11(2):e0147973. doi: 10.1371/journal.pone.0147973 (PMC4734623; doi:10.1371/journal.pone.0147973)
Supplement: S3 Table — (PDF) [file pone.0147973.s007.pdf]

Table S3. Genomic characterisation of the marker loci

Marker	Chromosome	CpG-Island	Gene symbol	Accession number (GenBank)	Gene symbol	Locus proximity
Blut1	16	no (63% GC) 8cg/344bp	C16orf54	Q6UWD8	Transmembranprotein c16orf54	Exon: 16orf54-001
Blut2	16	no (47% GC) 7cg/232bp	RAB11FIP3	O75154	Rab11 Family Interacting Protein	5´UTR: RAB11FIP3
Mens1	12	yes (69% GC) 25cg/293bp	SLC26A10	Q8NG04	Solute Carrier Family 26 Member 10 Pseudogene (Chloride/bicarbonate exchanger)	5´UTR: SLC26A10 in the region of nonsense-mediated mRNA decay
Spei1	2	no (58% GC) 13cg/211bp	SOX11	P35716	Transcription factor Sox11	332500bp-5´UTR: SOX11
Spei2	10	no (55% GC) 9cg/292bp	WBP1L/ OPA1L	Q9NX94	WW domain binding protein 1-like	3´-site: Exon WBP1L-001 (OPA1L, C10orf26)
Vag1	2	yes (58% GC) 20cg/272bp	HOXD4	P09016	Sequencespecific transcription factor HXD4	Exon: HOXD4-001
Vag2	2	yes (57% GC) 8cg/188bp	HOXD12	P35452	Sequencespecific transcription factor HXD12	74bp-5´UTR: HOXD12-001
Sperm1	6	no (66% GC) 13cg/217bp	TCP11	Q8WWU5	T-complex protein 11 homolog	5´-Ende: Exon TCP11-009
Sperm2	2	yes (62% GC) 7cg/215bp	VAMP8	Q9BV40	Vasicle-Associated Membrane Protein 8	158bp-3´UTR: VAMP8-003
